# Time-Dependent Regulation of IL-2R α-Chain (CD25) Expression by TCR Signal Strength and IL-2-Induced STAT5 Signaling in Activated Human Blood T Lymphocytes

**DOI:** 10.1371/journal.pone.0167215

**Published:** 2016-12-09

**Authors:** Alla N. Shatrova, Elena V. Mityushova, Irina O. Vassilieva, Nikolay D. Aksenov, Valery V. Zenin, Nikolay N. Nikolsky, Irina I. Marakhova

**Affiliations:** Department of Intracellular Signaling and Transport, Institute of Cytology, Russian Academy of Sciences, St-Petersburg, Russia; Swedish Neuroscience Institute, UNITED STATES

## Abstract

The expression of the IL-2R α-chain (IL-2Rα) is regulated at the transcriptional level via TCR- and IL-2R-signaling. The question is how to precede in time the activation signals to induce the IL-2Rα expression in native primary T cells. By comparing the effects of selective drugs on the dynamics of CD25 expression during the mitogen stimulation of human peripheral blood lymphocytes, we identified distinct Src- and JAK-dependent stages of IL-2Rα upregulation. PP2, a selective inhibitor of TCR-associated Src kinase, prevents CD25 expression at initial stages of T cell activation, prior to the cell growth. This early IL-2Rα upregulation underlies the T cell competence and the IL-2 responsiveness. We found that the activated with “weak” mitogen, the population of blood lymphocytes has some pool of competent CD25+ cells bearing a high affinity IL-2R. A distinct pattern of IL-2R signaling in resting and competent T lymphocytes has been shown. Based on the inhibitory effect of WHI-P131, a selective drug of JAK3 kinase activity, we concluded that in quiescent primary T lymphocytes, the constitutive STAT3 and the IL-2-induced prolonged STAT5 activity (assayed by tyrosine phosphorylation) is mostly JAK3-independent. In competent T cells, in the presence of IL-2 JAK3/STAT5 pathway is switched to maintain the higher and sustained IL-2Rα expression as well as cell growth and proliferation. We believe that understanding the temporal coordination of antigen- and cytokine-evoked signals in primary T cells may be useful for improving immunotherapeutic strategies.

## Introduction

T cell activation involves two major steps of signal transduction events. T cell receptor (TCR) complex upon specific antigen recognition initiates the first signal that regulates the expression of specific genes, including cytokines and cytokine receptors [1–3]. TCR-induced expression of interleukin-2 (IL-2) and IL-2 receptor α-chain (IL-2Rα) starts the second wave of signaling events that, ultimately, result in T cell proliferation through activation of diverse target genes[4, 5]. Among other activation events, the expression of the IL-2Rα regulates the magnitude of T cell proliferative response. The expression of the IL-2Rα gene assists the formation of high affinity receptor for IL-2 through the association of the α-chain with two polypeptide chains, IL-2Rβ and IL-2Rγ, which are constitutively expressed at the surface membrane of quiescent T cells [6–8]. IL-2Rα expression increases the affinity of IL-2 binding ~100 times, facilitating IL-2 responses at low physiological concentrations of IL-2 [9–11]. Compromised expression of IL-2 or IL-2Rα leads to the development of autoimmune diseases and immunodeficiency [12–14].

Expression of the IL-2Rα is tightly regulated at the transcriptional level. Several positive regulatory regions control activation-dependent IL-2Rα induction in response to antigen and IL-2 [9, 15]. The current concept of IL-2Rα gene induction in T cell requires a coordinated effort between signaling pathways downstream of the TCR and the IL-2R. Although much is known about the molecular mechanisms that results in the IL-2Rα upregulation, some questions remain. To test cooperation between TCR and IL-2R downstream signaling the transgenic mouse models and IL-2R-deficient T cells have been widely used with the assumption that intracellular signaling in these cells would be identical to that in normal T cells. Nevertheless, there is evidence that in knockout models other signaling may be engaged in cell activation that compensates the signaling switch off in cells in vivo [16, 17]. Little studies are available on intact primary T cells, and how antigen- and cytokine-evoked signals are timely coordinated under physiological conditions to induce the IL-2Rα expression is poorly investigated. Meanwhile, the induction of the functional system composed of IL-2 and high affinity IL-2R is critical for T cell proliferation and the effective immune response.

In the present study, we addressed this question. The IL-2Rα expression was assessed by flow cytometry analysis of CD25 which is a cell surface marker of IL-2Rα. At first, using selective kinase inhibitors we identified Src- and JAK-dependent stages of CD25 expression in mitogen-stimulated human blood T lymphocytes. Further, we established the crucial role of initial TCR-signaling for IL-2Rα expression and emphasized that it is in competent PBL having high-affinity IL-2R that the sustained JAK3/STAT5 signaling is switched on to provide the higher and long-term IL-2Rα upregulation.

## Materials and Methods

### Lymphocyte isolation and stimulation

Human peripheral blood lymphocytes (PBL) were isolated from fresh venous blood of healthy adult donors (collected with written consent with approval from The Institute of Cytology RAS and The State Institution “Mariinsky Hospital”, Saint-Petersburg, permission number 2025/14). PBL were obtained by density gradient centrifugation over Histopaque (Histopaque-1077, Sigma), followed by monocyte/macrophage depletion via plastic adherence [18].

Prior to experiments, purified cells were suspended at a concentration of 2x106 cells/ml, and allowed to rest overnight in RPMI medium supplemented with 5% heat-inactivated human serum (AB IV Rh+). At the next day the cell suspension (>85% CD3^+^ cells) were distributed into plates (10-20x10^6^ cells/plate) and stimulated either with the polyclonal mitogen for T lymphocytes phytohemagglutinin (PHA-M, Sigma, USA) or with human recombined IL-2 (Biotex, Russia) in the absence or presence of pharmacological inhibitors WHI-P131, 4-(4´-Hydroxyphenyl)amino-6,7-dimethoxyquinozoline or PP2, 4-Amino-5-(4-chlorohyenyl)-7-(t-butyl)pyrazolo[3,4-d]pirimidine (Calbiochem, USA), or left unstimulated.

### Flow cytometry

The relative levels of CD4 and CD25 expression as well as the proliferation of cultivated PBL were assessed by flow cytometry. PBL were pelleted by centrifugation, rinsed once and suspended in PBS (10^6^ cells/ml). Cells were stained with fluorescein isothiocyanate (FITC)-labeled CD25 Abs and with phycoerythrin (PE)-labeled CD4 Abs (Invitrogen, USA). Mouse IgG-FITC and IgG-PE isotype control were used for assessing the background staining of cells. The percentage of CD25+ or CD4+ cells was determined after gating on lymphocytes. Cells were analyzed on a Coulter Epics XL Flow Cytometer (Beckman Coulter, Brea, CA, USA). Two parameter histograms were used (CD4LOG versus FSLOG, CD25LOG versus FSLOG as well as CD4LOG versus CD25LOG). Cell enlargement of induced PBL or drug inhibitory effect on growth of PBL was also seen on the two parameter histograms. The dot plots were obtained with WinMDI Version 2.8. Statistical analysis of results was performed according to the standard protocol of the data treatment EpicsXL.

To assess the proliferative response of stimulated PBL, at definite time after mitogenic stimulation, PBL (10^6^ cells/ml) were suspended in PBS containing 200 μg/ml of saponin (Sigma) for 30 min, then washed and treated with 250 μg/ml ribonuclease (Serva, Germany) and 50 μg/ml propidium iodide (Sigma, USA) in PBS for 30 min at 37°C. Cell cycle distribution was analyzed by Coulter Epics XL Flow Cytometer (Beckman Coulter, Brea, CA, USA). Cells were classified as G_0_/G_1_/ S and G_2_/M. List mode files were analyzed with ModFit LT software (Verity Software House, Topsham, ME, USA).

### Western blotting

Total cell lysates were prepared as described previously [19]. PBL were pelleted by centrifugation, washed twice in PBS, suspended in lysis buffer TBS (50 mM Tris-HCl, 1% Ttriton X-100, 0.5% nonidete P-40, 150 mM NaCl, 1mM EDTA, protease and phosphatase inhibitors), homogenized and centrifuged at 15,000 g (10 min). All manipulations and solutions were at 4°C. Total protein was quantified using the Bradford assay. SDS-PAGE electrophoresis, transfer to nitrocellulose membrane and immunoblotting with ECL (Thermo Scientific, USA) detection was performed according to standard manufacturer’s protocols (Bio-Rad Laboratories, USA). Phospho-STAT3 (P-STAT3) and phospho-STAT5 (P-STAT5) levels were assessed by Western blotting overnight at 4°C with P-STAT3 and P-STAT5 antibodies. Membranes were washed with TTBS (20 mM Tris-HCl, pH 7.5, 150 mM NaCl, 0.1% Tween-20), incubated with 5% skim milk in TTBS for 1h, then with primary antibodies in 5% BSA (bovine serum albumin) in TTBS for 10−12 h, washed with TTBS, incubated with secondary antibodies in 5% skim milk in TTBS for 1 h and washed with TTBS. Bands were detected with HRP-conjugated anti-rabbit or anti-mouse Abs and bound Abs were detected by ECL (Amersham). When reblotting, membranes were cleaned in stripping buffer, blocked in TBS with 1% BSA and reprobed for STAT3 or STAT5 with appropriate Abs. Blots were scanned by Epson perfection (4490PHOTO) and quantified with Scion image software. For quantitating western blots, we take lower exposure images where the bands or signals are not saturating. And always quantitate two or three different exposures and compare among them whether they are giving values close. At each time point P-STAT3 and P-STAT5 levels relative to STAT3 and STAT5 levels were calculated and these values were normalized to those from samples at 24 h on the same protein gel blot to determine fold change.

The following antibodies were used: phospho-STAT3 (Tyr705), phospho-STAT5 (Tyr694), phospho-JAK3 (Tyr980/981), STAT3, STAT5, JAK3. All antibodies were purchased from Cell Signalling, USA. Antibodies (1:1000) were diluted with 5% BSA in 0.1% TTBS. Secondary antibodies to these primary antibodies: anti-rabbit goat antibodies conjugated with horseradish peroxidase (Cell Signalling, United States), dilution 1:6000. β-Actin was assessed as a loading control: monoclonal antibodies to β-actin were diluted 1:2000 with 1% BSA. Secondary antibodies to these monoclonals: anti-mouse goat antibodies conjugated with horseradish peroxidase. β-Actin mouse mAbs (8H10D10) as well as HRP-linked anti-rabbit IgG were from Sigma-Aldrich, USA.

### RT-PCR assay

Total RNA from PBL was extracted by guanidine thiocyanate method [20]. 2–3μg RNA was reverse-transcribed (MMLV Gibco BRL) using 1μg of random hexamers in a volume 20 μl. Primers for IL-2Rα (forward 5`-CCACTCGTCCTGGGACAACC-3`, reverse 5`-CATATGAGCTGGGGCTGGGTC-3`, 336 bp) were synthesized according to the sequences published previously [21]. Housekeeping gene β-glucuronidase (GUS) was used as an internal control. Primers for GUS were as follows: forward 5`-GAAAATACGTGGTTGGAGAGCTCATT-3`, reverse 5`-CCGAGTGAAGATCCCCTTTTTA-3`, 101 bp. All the primers were synthesized by Sintol (Russia). To avoid false positive results due to genomic contamination of the samples, the primers spanned an intron at the genomic level. PCR was carried out in a volume 10 μl using 2 μl diluted cDNA, 0.5 μM of each primer, 0.2 mM each of dNTP, 1.4 mM MgCl_2,_ 1 × Hot-*Taq* polymerase buffer (Sileks, Russia) and 1 U Hot-*Taq* polymerase. Amplification conditions were 95°C 10 min, then 25 cycles (for GUS) or 26 cycles (for IL-2Rα) at 94°C 40s, 58°C 40s, 72°C 40s. All reactions were performed in triplicate at a minimum. The resulting DNA products were electrophoresed on a 6% polyacrylamide gel, stained in ethidium bromide, visualized by UV fluorescence and quantitated using NIH Image J software. All the results of PCR-RT analysis were obtained on PBL from five donors.

### Assessment of cell viability

Propidium iodide (PI) staining was used to determine cell viability after drug treatment. PI is excluded by viable cells but can penetrate cell membranes of dead cells and intercalates into double-stranded nucleic acids. PI (0.05 mg/ml) was added to the samples just before analysis, mixed gently and analyzed by flow cytometry (Beckman Coulter). Each sample was analyzed for 50 s and at least 20,000 cells were acquired for analysis. Triplicate counts were obtained for each procedure.

The cell viability was also assessed by measuring intracellular monovalent cation content. In experiments, in parallel probes (5x10^5^ cells), untreated or treated with drugs, the cell potassium (K_i_) and sodium (Na_i_) contents were determined by flame emission photometry (Perkin-Elmer AA306 spectrophotometer) as described previously [18]. The high and constant K_i_ / Na_i_ ratios were considered as the physiological marker for functional viability of cultivated PBL [22].

### Statistical analysis

Graphs shown throughout the paper show the mean and standard error of the mean from several independent experiments performed. When appropriate, statistical differences were calculated using the Student’s *t*-test and considering significant at P <0.05.

## Results

### The long-term CD25 expression is regulated by two independent signals via TCR and high-affinity IL-2R in human PBL

**Fig 1:**
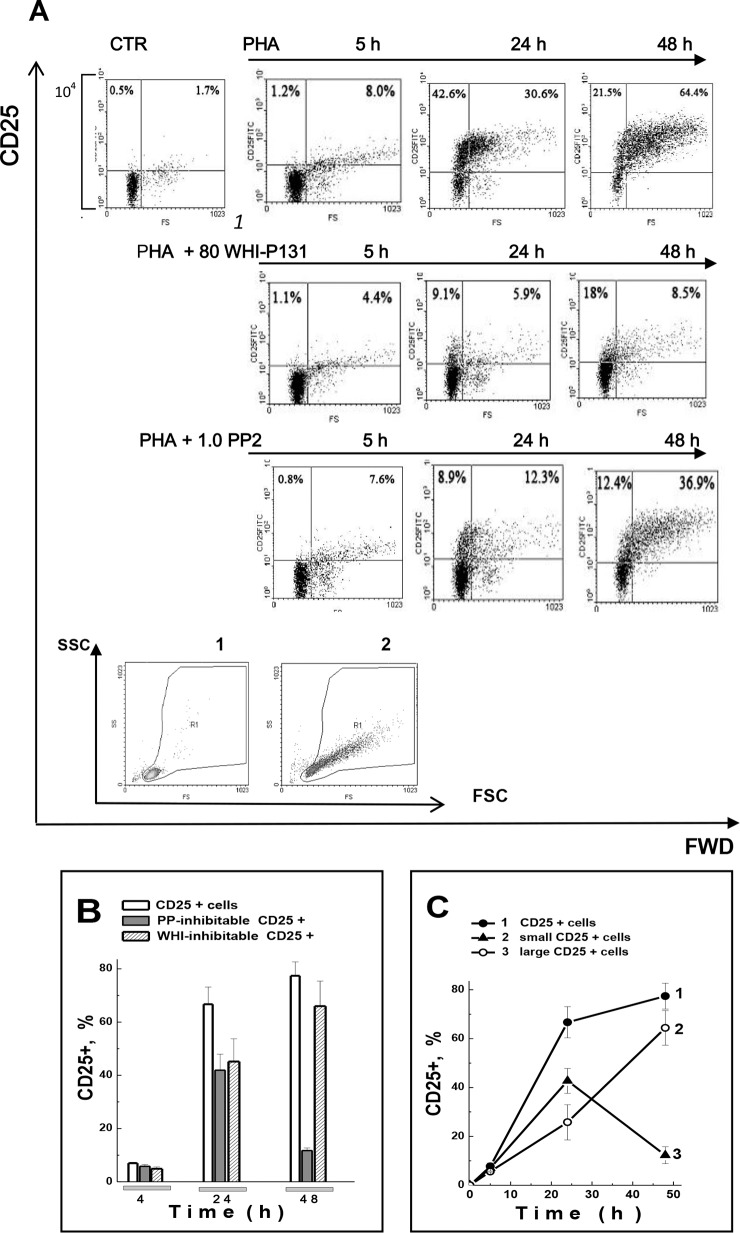
PHA stimulation leads to long-term CD25 expression in human blood T lymphocytes. (A) The representative histograms of nine experiments on PBL from different donors are shown. PBL were cultivated with 10 μg/ml PHA in the absence or presence of 80 μM WHI-P131 (WHI) or 1.0 μM PP2 and after 5, 24 or 48 h were analyzed by flow cytometry. The dot plots have been obtained with Win MDI Version 2.8. In order to identify the axis labels, the additional vertical axis is shown in the upper row of histograms. Y-axis is 4 decades logarithmic scale. **1, 2 –**The gate was chosen considering the cell size increase during blasttransformation: 1- CTR—control, non-stimulated PBL, 2 –after 48 h of culture with 10 μg/ml PHA. X-axis–forward scatter (FSC), Y-axis–side scatter (SSC). (B) Time-course of CD25+ expression in PHA-induced PBL. 1 –Total number of CD25+ cells, 2—large CD25+ cells, 3—small CD25+ cells. (C) WHI-P131- and PP2-inibitable portions of CD25+ cells in PHA-stimulated PBL. Summary of independent experiments is presented as mean ±SEM (n = 9, p˂0.05). In each experiment, when analyzing the PHA-induced CD25 expression changes, the background CD25 expression in resting cells (CTR) was subtracted. Summary data are presented as mean ± SEM (n = 10, p˂0.05).

In previous studies, using selective pharmacological inhibitors we discriminated Src- and JAK-dependent stages in the course of CD25 expression in activated human peripheral blood T lymphocytes (PBL) [23, 24]. In this work, we studied time- and dose-dependent relations between drug-inhibitable portions of CD25 expression and present new evidence that under physiological conditions in quiescent human T cells, induction of IL-2Rα is timely regulated by two independent signals via TCR and high-affinity IL-2R. Fig 1 presents the summary results of these experiments. Freshly isolated PBL were treated with phytohemagglutinin (PHA, 10 μg/ml). PHA, a polyclonal mitogen for T lymphocytes, induces structural changes in TCR/CD3 complex, triggers the cascade of signaling events that characterizes the early stage of antigen activation, and mitogen doses of PHA induce the T lymphocytes exit from quiescence into the cell cycle [1, 4, 25]. PHA was added to PBL alone or in the presence of selective tyrosine kinase inhibitors. We applied PP2 (4-Amino-5-(4-chlorohyenyl)-7-(t-butyl)pyrazolo[3,4-d]pyrimidine) as an inhibitor for the Lck kinase. PP2 is at least 1,000-fold more active against Src family kinases than other TCR-associated tyrosine kinases [26, 27]. It has also been shown that PP2 is effective in blocking the anti-CD3-induced T-cell activation events, while it is less effective at inhibiting the TCR-independent proliferation induced by phorbol ester and IL-2 [28]. To determine directly whether IL-2R signaling is necessary for CD25 expression in intact T-lymphocytes, we used WHI-P131 (4-(4´-Hydroxyphenyl)amino-6,7-dimethoxyquinozoline) as an effective inhibitor of IL-2R-associated tyrosine kinase JAK3. WHI-P131 did not inhibit JAK1 and JAK2, the Src and Tec families tyrosine kinases [29].

In PBL populations obtained from different healthy individuals CD25 could be detectable in 0.5–6% of the total T cell pool. These CD4^+^CD25^+^ cells appear to represent the CD4+CD25+ T regulatory cells (natural Tregs) [30, 31]. The increased CD25 expression was detectable at 5 h after PHA addition (Fig 1A, top row). Later, the percentage of CD25+ cells was substantially increased from (8.7±0.8%, n = 10) after 5 h to (76.9±7.4%, n  =  10) at 48 h, and to the end of the second day only, the PBL population becomes represented mainly by large CD25+ cells (Fig 1B and 1C). Both PP2 and WHI-P131decreased the number of CD25+ cells in PHA-induced cultures, reduced the S-phase induction and suppressed the growth of activated PBL (Fig 1A; Table 1). As follows from PI staining test in concentrations used PP2 (up to 1 μM) and WHI-P131 (up to 80 μM) did not increase significantly the number of dead cells in culture (Table 1). Drug-treated PBL maintained also the normal ion homeostasis (as judged by high potassium and low sodium content in cells) and were functionally viable (Table 1).

**Table 1:** The growth, proliferation and viability of human blood T lymphocytes, stimulated with PHA or IL-2 in the absence or presence of WHI-P131 and PP2 for 48 h.

Incubation	Cell growth ng / 10^6^cells	Proliferation S+G_2_+M, %	Ion ratio K_i_ /Na_i_	Cell death %
Resting PBL	52 ± 3±	1.1 ± 0.02	4.3 ± 0.75	5.3 ± 0.7
PHA10	133±14	43.7 ± 2.5	5.1 ± 0.78	13.7 ± 1.0
PHA10 +WHI-P131, 50	84±7	14.7 ± 1.5	4.9 ± 0.90	15.0 ± 0.7
PHA10 +WHI-P131, 80	64±5	5.4 ± 0.2	4.3 ± 0.58	15.8 ±1.0
PHA10 + PP2, 1.0	68 ± 7	10.4 ± 0.9	4.5 ± 0.70	12.1 ±0.7
IL-2, 100	60 ± 4	2.3 ± 0.1	4.3 ± 0.60	3.2 ±0.3
PHA 0.7	50 ± 4	6.9 ± 0.1	4.4 ± 0.70	5.9 ±0.6
PHA 0.7+IL-2, 100	119 ± 13	30.6 ± 5.4	5.0 ± 0.47	6.1 ±0.6

Isolated PBL were cultured 48 h with 10 μg/ml PHA in the absence or presence of 50 or 80 μM WHI-P131 or 1.0 μM PP2, or isolated PBL were incubated with submitogenic PHA (0.7 μg/ml) for 20 h and then IL-2 (200 U/ml) was added for the next 24 h. Cell growth was evaluated from protein measurements by Lowry method in the same probes where intracellular ion content (K_i_/Na_i_) was measured. The percentage of cells in (S+G_2_+M) phases was determined from cell cycle analysis by flow cytometry. The high K_i_/Na_i_ ratio was considered as the physiological marker for functional viability of PBL. Cell death was assessed by propidium iodide (PI) incorporation into cells. The results indicate the percentage of the PI-staining cells as determined by FASC analysis. Data represent mean ± SD of five independent experiments with PBL from different donors.

We revealed that the PP2-inhibitable component of CD25 expression in PHA-stimulated PBL was more significant in the small CD25+ cells, which number was peaked at the end of the first day of activation (Fig 1A and 1B). During the second day, PP2-inhibitable expression of CD25 reduced (Fig 1C). Unlike PP2 the inhibitory effect of WHI-P131 was observed during the whole transit of activated PBL to proliferation and after 48 h up to 90% of large blasts had CD25 markers.

**Fig 2:**
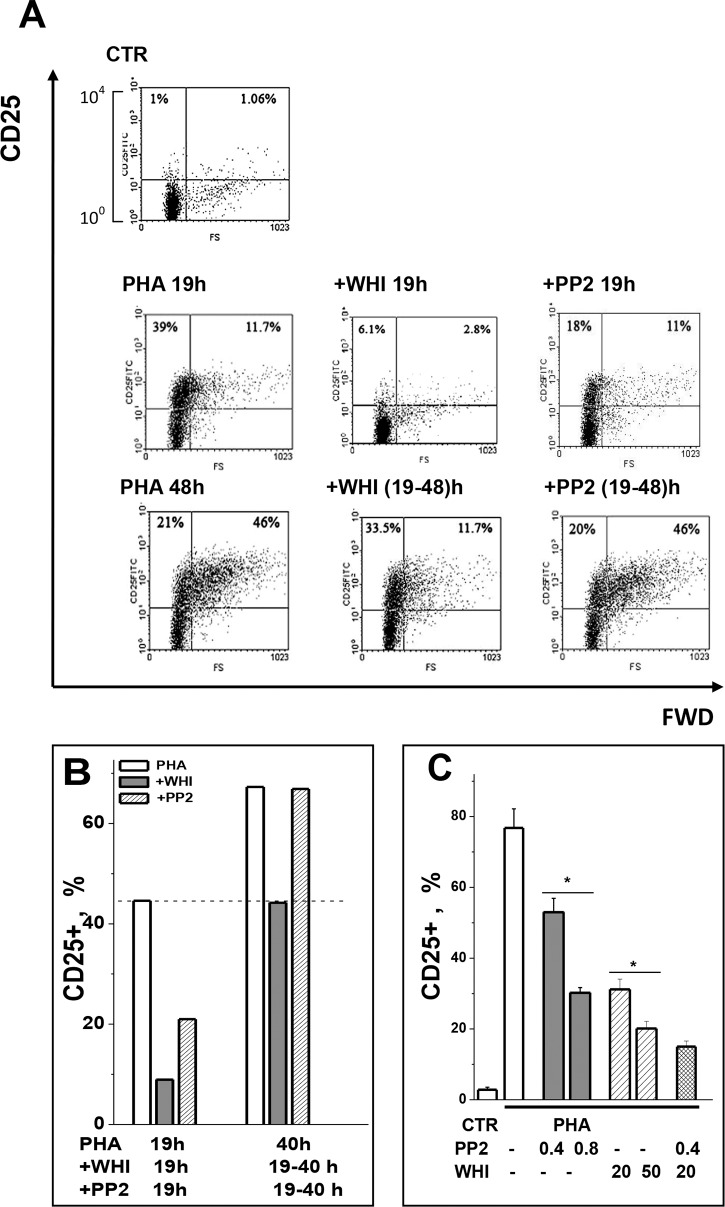
The inhibitory effect of WHI-P131 and PP2 on CD25 expression in PHA-stimulated human blood T lymphocytes. (A) The PP2-inhibitable expression of CD25 is timely associated with the initial stages of PBL activation. Cells were cultivated with 10 μg/ml PHA without or with 80 μM WHI-P131 (WHI) or 1.0 μM PP2 for 19 h (middle row) or cells were cultivated with 10 μg/ml PHA for 19 h and thereafter WHI-P131 or PP2 were added for the next 21 h (bottom row). Additional Y-axis is 4 decades logarithmic scale. Representative data on PBL from one donor are shown. (B) Summary data of four independent experiments are shown as mean + SEM (n = 4, p˂0.05). (C) The inhibitory effects of WHI-P131 (WHI) and PP2 on CD25 expression are additive. Cells were cultivated with 10 μg/ml PHA without or with WHI-P131 (WHI, 20 and 50 μM), or PP2 (0.4 and 0.8 μM), or WHI-P131 (20 μM) and PP2 (0.4 μM) were given simultaneously for 24 h. Summary of independent experiments are presented as mean + SEM (n = 4). *, *p<*0.05. CTR—control, non-stimulated PBL.

In order to clarify whether the inhibitory effect of PP2 depends on the time of cell activation, we compared the drug effect at different time points of PHA stimulation and found that during the first 19 h, PP2 prevented increase in the number of CD25+ cells while reducing CD25 expression on small cells (Fig 2A and 2B). When applied 19 h after PHA addition PP2 had no effect on the increase in number of CD25+ cells which occurred during the second day of stimulation (Fig 2B). The effects of PP2 and WHI-P131 on CD25 expression were additive (Fig 2C). Altogether our data indicate that (1) PP2-inhibitable expression of CD25 is associated with the early stages of mitogen-induced PBL activation and precedes the cell growth and (2) TCR- and IL-2R-evoked downstream signals must cooperate in time to provide the sustained IL-2Rα upregulation.

### IL-2 up-regulates the CD25 expression in competent but not in quiescent PBL

**Fig 3:**
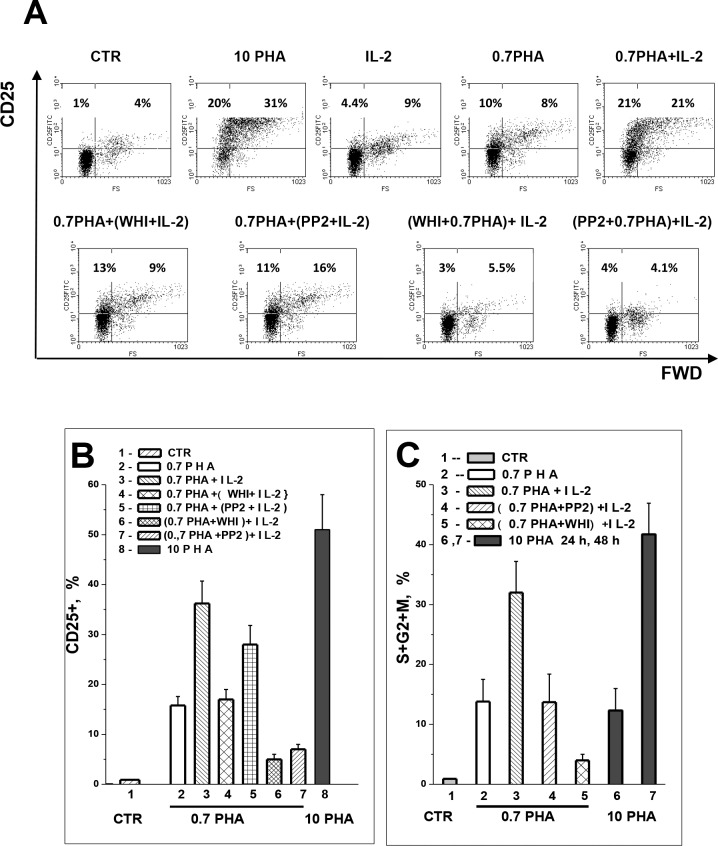
Stimulation with non-mitogenic PHA and IL-2 leads to long-term CD25 expression and proliferation in human blood T lymphocytes. (A) The representative histograms of one experiment on PBL from one donor are shown. PBL were not stimulated (CTR) or stimulated with 0.7 μg/ml (0.7PHA), or 10 μg/ml (10PHA) PHA, or 200 U/ml IL-2 (IL-2) for 48 h. In the same experiment, PBL were preincubated in culture medium with 0.7 μg/ml PHA and 80 μM WHI-P131 (WHI) or 1.0 μM PP2 for 24 h prior to IL-2 stimulation, or drugs were added simultaneously with 0.7 μg/ml PHA for 24 h and thereafter IL-2 was added for the next 24 h. Additional Y-axis is 4 decades logarithmic scale. (B) CD25 expression in resting PBL in response to 0.7 μg/ml PHA (2) and 10 μg/ml PHA (8), or in competent PBL in response to IL-2 in the absence (3) or presence of WHI-P131 (4) or PP2 (5), or in response to IL-2 in PBL preactivated with 0.7 μM PHA in the presence of WHI-P131 (6) or PP2 (7). Summary data of independent experiments on PBL from different donors are shown as mean + SEM (n = 4, p˂0.05). (C) Proliferation of resting PBL in response to 0.7 μg/ml PHA (2) and 10 μg/ml PHA (6, 7), or competent PBL in response to IL-2 in the absence (3) or presence of PP2 (4) or WHI-P131 (5). Bars represent the number of cells in (S+G2+M) phases of cell cycle at 48h after PBL stimulation. Summary data of independent experiments on PBL from different donors is shown as mean + SEM (n = 4, p˂0.05).

To make clear the role of early PP2-inhibitable CD25 induction we investigated whether exogenous IL-2 can induce the expression of IL-2Rα in quiescent T cells and found that in freshly isolated PBL, IL-2 failed to induce a full proliferative response (4.3% cells in S+G_2_+M-phases as compared to 43,7% in PHA-stimulated PBL cultures after 48 h) (Table 1). IL-2 also was not capable inducing higher CD25 expression in resting PBL: in IL-2 stimulated cultures (50–250 U/ml, 48 h) the number of large CD25+ cells did not change or increased to no more than 9.6% (Fig 3A).

It has previously been shown that incubation of quiescent T cells with low doses of PHA that did not induce cell proliferation, render the cells responsive to IL-2 [32]. We used this experimental protocol and revealed that activated with “weak”, 0.7 μg/ml PHA, PBL cultures contain increased number of CD25+ cells, 15.8±1,8% (n = 4) (Fig 3A and 3B, column 2). Remarkably, this population of CD25+ cells which should bear on the surface high-affinity IL-2R was present in PBL culture during the whole observation period (48 h). Further, in “conditioned”, competent PBL, IL-2 increased surface CD25 expression: the number of CD25+ cells in competent PBL after 48 h stimulation with IL-2 was comparable with the number of CD25+ cells in PBL that were stimulated with the “strong”, 10 μg/ml PHA (Fig 3B, columns 3 and 8). In addition, competent cells enter into the cell cycle and proliferate well in the presence of IL-2 (Fig 3C, column 3; Table 1). In view of the above data it is reasonable to assume that the ability of IL-2 to promote IL-2Rα expression and cell proliferation in competent (but not in quiescent) PBL cultures is provided by a pool of CD25+ cells with the functionally active IL-2R having α-chain.

To identify signals that might be involved in competence induction, we assessed the effect of kinase inhibitors on CD25 expression. In one set of experiments competent PBL had been treated with WHI-P131 or PP2 for 1 h before IL-2 was added for the next 24 h. We revealed that WHI-P131 totally inhibited CD25 expression in response to IL-2 in competent PBL (Fig 3B, column 4). Under the same conditions, the IL-2-induced CD25 expression was attenuated in the presence of PP2 by no more than 40% (Fig 3B, column 5). In another set of experiments the drugs were given during the preactivation, i.e. simultaneously with “weak” PHA. Under these conditions, PP2 strongly inhibited the CD25 expression in response to IL-2 (Fig 3B, column 7). These data suggest that the competence induction and priming the quiescent T cells to IL-2 can be provided by Src-associated signaling via “weak” TCR stimulation.

**Fig 4:**
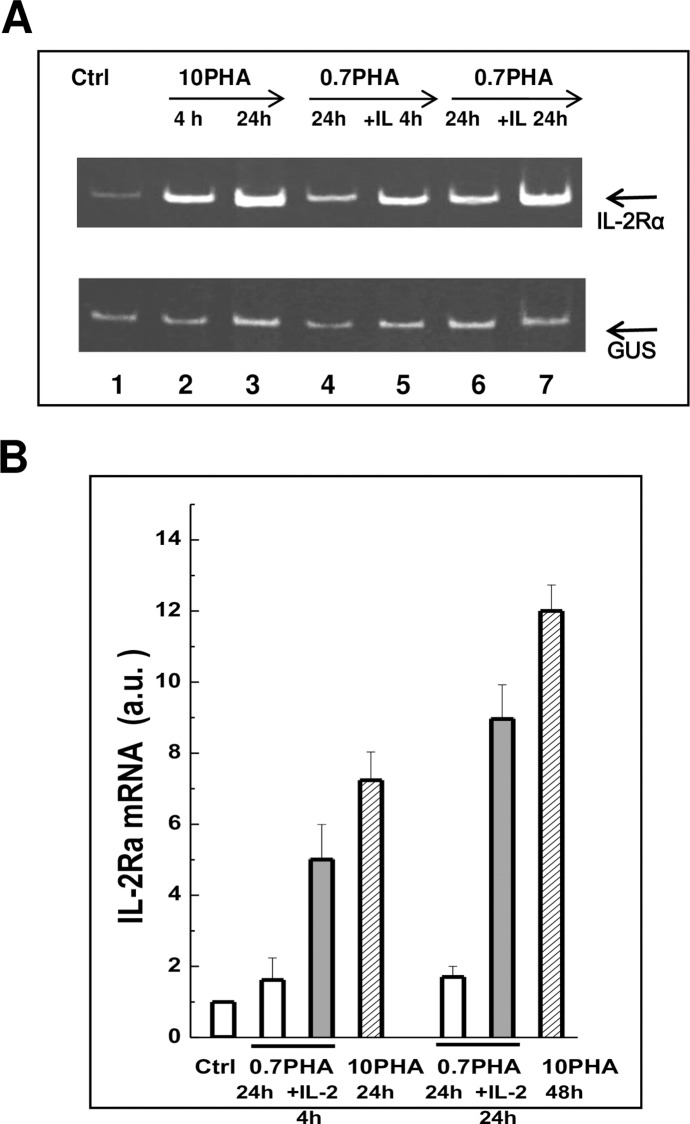
Expression of mRNA encoding for IL-2Rα in human blood T lymphocytes stimulated by PHA or IL-2. (A) The representative PCR amplification of IL-2Rα in PBL, resting (Ctrl) or stimulated with 10 μg/ml PHA (10PHA) for 4 and 24 h, or in competent lymphocytes treated with 0.7 μg/ml PHA (0.7PHA) for 24 h and then stimulated by IL-2 (0.7PHA+IL-2) for 4 and 24 h. β-glucuronidase (GUS) was used as an internal control. (B) Quantification of the results presented in (A). The mRNA expression is shown relative to the optical signals obtained with the RNAs in resting or competent PBL. The summary data of independent experiments on PBL of four donors, mean ± SEM (n = 4).

To find out whether there is a difference in the regulation of IL-2Rα expression in competent and mitogen-stimulated PBL we evaluated the content of IL-2Rα transcripts by RT-PCR analysis. As shown in Fig 4A, in resting PBL, IL-2Rα mRNA was barely detectable. After PBL stimulation with 10 μg/ml PHA the level of IL-2Rα mRNA rose within 2 h and increased gradually to the high level for the next 24 h (Fig 4B). On the contrary, incubation of PBL with “weak” 0.7 μg/ml PHA was accompanied by 1.5-2-fold increase in IL-2Rα mRNA level, which was maintained low during two days (Fig 4B). Remarkably, after IL-2 stimulation of competent PBL, the IL-2Rα mRNA content was increased within 2–4 h to the level in PBL stimulated with “strong” mitogen concentration of PHA.

### Relations between STAT5 activation and CD25 expression in human activated with IL-2 or PHA

**Fig 5:**
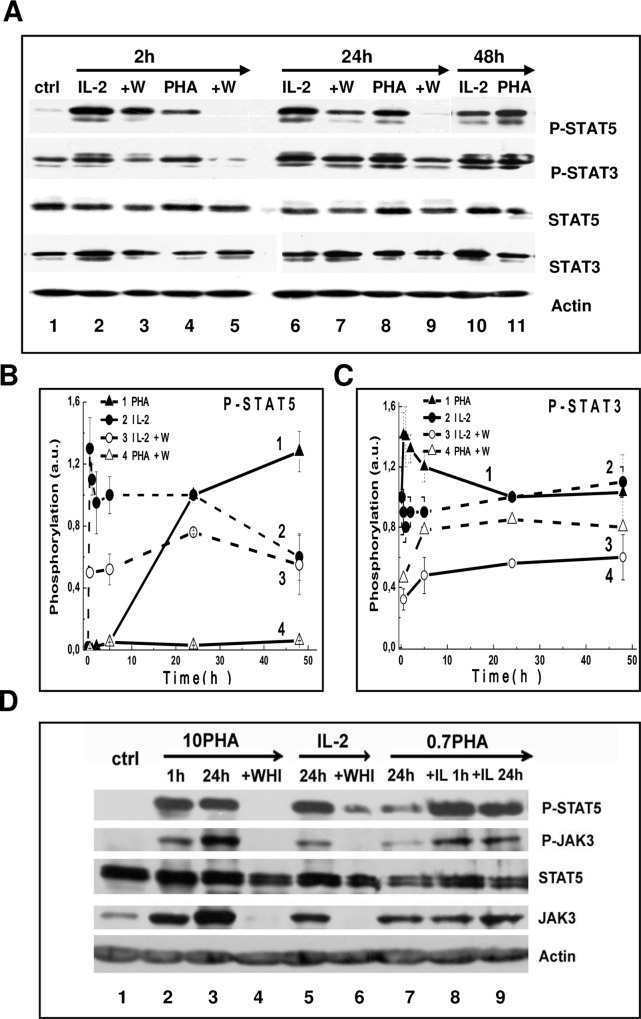
PHA induces delayed and sustained STAT5 activation in human blood T lymphocytes. (A) Representative Western blots of total lysates of PBL stimulated with 100 units/ml IL-2 (IL-2) or 10 μg/ml PHA (PHA) for 2, 24 or 48 h in the absence or presence of 80 μM WHI-P131 (W). (B) and (C) Densitometric analysis of STAT5 (B) and STAT3 (C) tyrosine phosphorylation in PBL stimulated with PHA (line 1) or IL-2 (line2) in the absence or presence of 80 μM WHI-P131 (W, lines 3 and 4). At each time point P-STAT5 and P-STAT3 levels relative to total STAT5 and total STAT3 levels were calculated and these values were normalized to those from samples at 24 h on the same protein gel blot to determine fold change. Summary of independent experiments on PBL from six donors are presented as mean ± SEM (n = 6, p˂0.05). Significant changes from P-STAT/STAT ratio at 24 h are indicated with an asterisk, p˂0.05. (D) Sustained STAT5 activity is provided by JAK3 kinase in mitogen-induced PBL. PBL were stimulated with 10 μg/ml PHA (10PHA) or with 100U/ml IL-2 (IL-2) or IL-2 after pretreatment of resting PBL with 0.7 μg/ml PHA (0.7PHA) in the absence or presence of 80 μM WHI-P131 (WHI). Data are representative of three experiments on PBL from different healthy donors. Ctrl—control, unstimulated PBL.

In order to clarify why IL-2 alone failed to induce the higher expression of CD25 and proliferation in quiescent PBL, we analyzed the STAT signals after IL-2 stimulation. In primary T cells, the intermediate affinity IL-2Rβγ_c_ is capable of signaling: heterodimerization of the IL-2Rβ and IL-2Rγ_c_ cytoplasmic domains is sufficient for IL-2 signal transduction defined as JAK and STAT phosphorylation [33, 34]. In this paper we compared IL-2-induced changes in STAT3 and STAT5 activities as the main IL-2R downstream signals and focused on STAT5 as the transcriptional factor which participate in IL-2Rα gene induction [7, 9]. Phospho-STAT5 (P-STAT5) was absent or barely detectable in unstimulated PBL (Fig 5A, lane 1). A high level of P-STAT5 was observed already in 1–2 h IL-2 addition (Fig 5A, lane 2). During the next 24 h the level of STAT5 phosphorylation remains high but further reduced (Fig 5C, line 2). Unlike STAT5, Phospho-STAT3 (P-STAT3) was always estimated in resting PBL (Fig 5A, lane 1). In response to IL-2 the content of P-STAT3 was transiently increased and then returned to the initial level (Fig 5C, line 2). These experiments indicate that IL-2 induces STAT5 activation in intact T lymphocytes however under these conditions STAT5 signal is attenuated with time.

WHI-P131, as an inhibitor of tyrosine kinase JAK 3, reduced the rapid IL-2-induced increase in P-STAT5 and P-STAT3 to about one third, however, at 24 h the inhibition of STAT phosphorylation was attenuated (Fig 5B, line 3 and 5C, line 3). This suggest that in quiescent, primary PBL, (1) IL-2-induced short-term regulation of both STAT3 and STAT5 activity may be provided by JAK3-dependent mechanism, whereas (2) the sustained STAT3 as well as long-term STAT5 phosphorylation in the presence of IL-2 appeared to be JAK3-independent.

Next, we examined the IL-2 action on the phosphorylation status of STAT proteins in competent PBL after preactivation with “weak” PHA. The phosphorylation status of STAT3 in competent cells was as high as in resting cells and IL-2 did not affect or slightly increased STAT3 phosphorylation but only during first 0.5–2 h (data not shown). In contrast, there was no or little phosphorylation of STAT5 in “conditioned” competent PBL (Fig 5D, lane 7). In competent cells, IL-2 rapidly increased the phosphorylation of STAT5 and later on the high level of STAT5 phosphorylation maintained for the next 24 h (Fig 5D, lane 7). Thus, in contrast to resting PBL, in competent T lymphocytes, IL-2 induced the higher and sustained STAT5 activation.

The next task was to analyze STAT signals in PBL stimulated with mitogenic, “strong” PHA which was capable to induce high and long-term CD25 expressions in primary T lymphocytes. We revealed that P-STAT5 appeared at 2–5 h following PHA stimulation, reached the high level at 24 h and remained high during the entire period of observation (48 h) (Fig 5B, line 1). In contrast, P-STAT3 slightly increased in first hours after PHA addition and then remained unchanged (Fig 5C, line 1). The differences between STAT3 and STAT5 signals were also revealed when we investigated the effect of WHI-P131 on changes in P-STAT3 and P-STAT5. In PHA-induced PBL, in the presence of WHI-P131 STAT3 phosphorylation was half suppressed, whereas STAT5 phosphorylation was abrogated (Fig 5B, line 4 and 5C, line 4). These data suggest that in PHA-induced PBL, JAK3 is involved in STAT5 activation throughout the transit from resting state to proliferation. To test this assumption, we assessed the phosphorylation of STAT5 and JAK3 in activated PBL. In resting PBL as well as in competent cells, activated with “weak” PHA (0.7PHA) for 24 h, phosphorylation of STAT5 or JAK3 was hardly detectable (Fig 5D, lanes 1 and 7). In the presence of “strong” PHA (10PHA), high phosphorylation of both proteins was observed between 2 and 24 h and WHI-P131 inhibited totally the sustained STAT5 and JAK3 phosphorylation (Fig 5D, lanes 3 and 4). We also revealed that WHI-P131 diminished significantly the level of total JAK3. For example, in PBL treated with high concentration of WHI-P131 (80 μM) total JAK3 was not detected (Fig 5D, lane 4).

**Fig 6:**
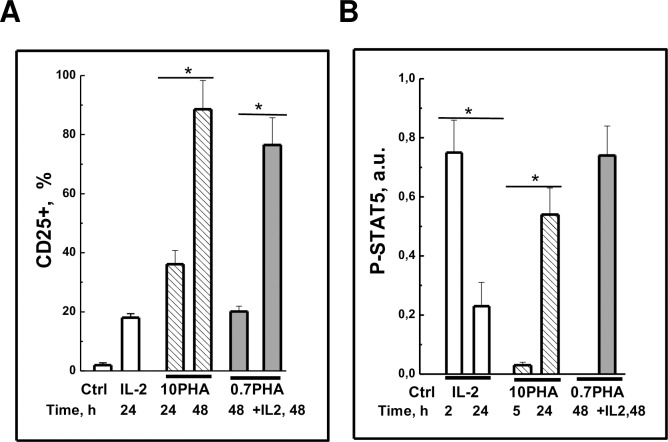
Sustained STAT5 phosphorylation is necessary to induce long-term CD25 expression in human T lymphocytes. (A) CD25+ cell number in PBL population stimulated with 100 units/ml IL-2 (IL-2) or 10 μg/ml PHA (10PHA) for 24 or 48 h or 0,7 μg/ml PHA(0.7PHA) for 48 h in the absence or presence of IL-2, %. Summary of independent experiments on PBL from four donors are presented as mean ± SEM (n = 4, p˂0.05). (B) Relative optical density in bands corresponding to normalized STAT5 tyrosine phosphorylation (P-STAT5). Summary of 11 independent experiments on PBL from different healthy donors are presented as mean + SEM (n = 11, p˂0.05). Ctrl—control, unstimulated PBL.

When comparing the experimental conditions for triggering of STAT5 phosphorylation, CD25 expression and proliferation in PBL, stimulated either with PHA or IL-2 in experiments on different donors, we found that the long-term surface expression of CD25 was always observed concurrently with the high and sustained STAT5 activity. Namely, in resting cells stimulated with strong, mitogenic PHA (10PHA) or in competent cells (0.7PHA) stimulated with IL-2 high number of CD25+ cells correlated with higher, sustained STAT5 phosphorylation (Fig 6A and 6B). In contrast, in resting cells stimulated with IL-2 there occurs short-term increase in STAT5 phosphorylation only and no CD25 expression.

Altogether, the above findings demonstrate that in primary human T cells exogenous IL-2 alone is capable to induce STAT5 signal however this signal is attenuated over time and is insufficient to induce the higher CD25 (IL-2Rα) expression and to start the cell cycle progression. To induce the high and prolonged CD25 expression that is associated with cell growth and proliferation, the quiescent T cells must be stimulated through the TCR by mitogen or the competent T cells may be stimulated by IL-2.

## Discussion

In this study, we distinguished pharmacologically distinct signaling pathways involved in IL-2Rα upregulation during the activation of human blood T lymphocytes. We have demonstrated that under physiological conditions in primary T cells, cell surface СD25 expression (used as a marker of IL-2Rα gene expression) is timely regulated via initial TCR signal strength and IL-2R-associated JAK3/STAT5 signaling. Data obtained are consistent with the two-step model of IL-2Rα expression in T cells [5, 9, 35]. TCR engagement by partial or full agonist initiates the first wave of gene expression, including IL-2Rα gene. This initial up-regulation of the IL-2Rα allows the formation of the trimeric IL-2Rαβγ_c_. Concomitantly, antigens also induce the secretion of IL-2, which in turn increase and prolong IL-2Rα expression via JAK3/STAT5 signaling. As a result, in the late activated T cells, there is a functional system IL-2/IL-2R that plays a critical role in T cell proliferation and effective immune response.

This model is based on detailed studies of transcriptional regulation of IL-2Rα gene expression. It is established that the transcription of IL-2Rα gene is controlled by six positive regulatory regions that are important for TCR-mediated regulation and for IL-2-mediated regulation of the gene [9, 15]. TCR-mediated activation of the IL-2Rα promoter involves the cooperation between several factors, including NF-kB, whereas IL-2-mediated regulation of the gene is mainly provided by STAT5.

Here, we have analyzed how the two-step model of IL-2Rα expression works in native primary T lymphocytes, stimulated with PHA as a polyclonal T cell mitogen. In order to distinguish distinct signaling pathways involved in IL-2Rα regulation in primary T cells we applied the selective drugs such as WHI-P131 and PP2. WHI-P131 is an effective inhibitor of IL-2R-associated tyrosine kinase JAK3 (29). PP2 is at least 1,000-fold more active against Src family kinases than other TCR-associated tyrosine kinases [26, 27]. In fact, PP2, a selective inhibitor of Src tyrosine kinase, prevents the downstream signaling pathways responsible for both IL-2 and IL-2Rα gene regulation. Therefore, it might be assumed that PP2 effects on CD25 expression was due to the decreased IL-2 production in the presence of PP2. However, as shown recently, T cell stimulation under experimental conditions in which IL-2 production was not induced or was prevented results in the induction of high-affinity IL-2R that was unable to transmit a proliferative signal [36].

Together our findings suggest that in primary peripheral T lymphocytes, stimulated through TCR complex by mitogen, IL-2-independent mechanism may be involved in early CD25 (IL-2Rα) expression. First, in quiescent primary PBL, exogenous IL-2 alone is not able to induce high CD25 expression which is typical for activated and proliferating T cells. Second, in quiescent primary PBL, stimulated by IL-2, STAT5 alone is not sufficient to induce the high and sustained CD25 expression and the proliferative response. Finally, PBL when treated with “weak” PHA has a small pool of competent CD25+ cells bearing a high affinity IL-2R. These competent cells are characterized by the elevated IL-2Rα mRNA expression, which was maintained at low but stable level throughout the “weak” PHA stimulation. In competent PBL STAT5 proteins, that can serve as markers of IL-induced cell cycle progression of T cells, are inactive. In the absence of IL-2 the competent cells remain “silent” and do not proliferate.

The transcription of IL-2Rα gene is controlled by six positive regulatory regions (PRRs) that are important for TCR-mediated regulation (PRRI and PRRII) and for IL-2-mediated regulation (PRRIII and PRRIV) of the gene [9, 15]. Of importance, PRRI binds NF-kB, whereas PRRIII and PRRIV bind STAT5A and STAT5B. Thus, TCR-mediated activation of the IL-2Rα promoter involves the cooperation between several factors, including NF-kB, whereas IL-2-mediated regulation of the gene is mainly provided by STAT5A and STAT5B. Based on these studies we suggest that in quiescent primary T cells, the initial signal for CD25 (IL-2Rα) expression may be directly provided by antigenic/mitogenic stimulation via TCR/Src/… NF-kB signaling pathway.

The effect of IL-2 in cellular immunity is mainly exerted by the transcription factors STAT via IL-2R-associated JAK1 and JAK3 tyrosine kinases [7, 33, 34, 37, 38]. STAT5 is uniquely required for the expression of genes that regulate the cell cycle in peripheral T cells [39]. Lack of STAT5 expression inhibits also the expression of CD25 leading to a failure of T cell proliferation [40]. As previously reported [41] and is also demonstrated in this study, in quiescent PBL, STAT5 proteins are inactive and exogenous IL-2 promotes STAT5 signal that alone is not sufficient to induce higher CD25 expression, blasttransformation and cell proliferation. Using chimeric receptors, it has been shown that a receptor containing the JAK boxes and one STAT5 docking site can mediate STAT5 activation but is unable to stimulate IL-2Rα expression [42]. It has also been reported that duration of STAT5 activation influences the response of IL-2Rα gene to cytokines [43].

Our experience shows that in primary T cells, the high. sustained CD25 expression takes place concurrently with the prolonged STAT5 activation. This occurs when “strong” PHA activates quiescent T cells or IL-2 stimulates competent T cells that were preactivated with “weak” mitogen. We next show that in competent T cells, IL-2 is able to turn on the sustained JAK3/STAT5 intracellular signaling. Indeed, it is assumed that the type of IL-2-induced JAK/STAT signaling may vary during the activation of T cells. As shown, in the absence of TCR stimuli, exogenous IL-2 induced survival signals from the intermediate affinity IL-2Rβγ_c_ and JAK3 is not involved in this signaling [44–47]. Here, based on the time dependency of CD25 expression and STAT5 phosphorylation in activated PBL, we suggest that in quiescent primary T cells, the prolonged STAT5 activity in the presence of IL-2 is mainly provided by JAK3-independent mechanism via IL-2R lacking CD25. It is interesting that JAK3 null mice do not generate CD25+CD4+ T cells [48]. On the contrary, in a population of “conditioned”, competent T cells bearing the СD25 in response to IL-2 JAK3/STAT5 signaling via the high-affinity IL-2Rαβγ_c_ is augmented to maintain the sustained IL-2Rα expression as well as cell growth and proliferation.

In summary, in this study we provide evidence for temporal relations between the initial mitogen-induced and the delayed IL-2-induced intracellular signaling involved in the IL-2Rα expression in human blood T lymphocytes. The role of preactivation is emphasized for cell surface expression of IL-2Rα. In the context of T cell activation, continuous STAT5 activity results in high T cell proliferation by an indirect mechanism, resulting from the IL-2Rα expression and STAT5 is essential for maximal responsiveness to antigens in vivo, underscoring the physiological importance of IL-2-induced IL-2Rα expression. Now, it is important to know whether signaling pathways downstream of the TCR and the IL-2R are also coordinated in time at the transcriptional level to induce the full program of IL-2Rα gene expression. Understanding the temporal relationships between antigen- and cytokine-evoked signals for the regulation of the IL-2R gene in human T cells may be useful for improving immunotherapeutic strategies.
